# Disinfection of the Access Orifice in NOTES: Evaluation of the Evidence Base

**DOI:** 10.1155/2011/245175

**Published:** 2011-07-12

**Authors:** Mikael H. Sodergren, Philip Pucher, James Clark, David R. C. James, Jenny Sockett, Nagy Matar, Julian Teare, Guang-Zhong Yang, Ara Darzi

**Affiliations:** ^1^Department of Biosurgery and Surgical Technology, Academic Surgical Unit, Imperial College London, St.Mary's Hospital, South Wharf Rd, Paddington W2 1NY, UK; ^2^Department of Interventional Endoscopy, Imperial College London, St.Mary's Hospital, South Wharf Rd, Paddington W2 1NY, UK; ^3^Institute of Biomedical Engineering, Imperial College London, South Kensington Campus, London SW7 2AZ, UK

## Abstract

*Introduction*. Appropriate prevention of infection is a key area of research in natural orifice translumenal endoscopic surgery (NOTES), as identified by the Natural Orifice Surgery Consortium for Assessment and Research (NOSCAR). *Methods*. A review of the literature was conducted evaluating the evidence base for access orifice preparation/treatment in NOTES procedures in the context of infectious complications. Recommendations based on the Oxford Centre for Evidence-Based Medicine guidelines were made. *Results*. The most robust evidence includes several experimental randomised controlled trials assessing infectious complications in the transgastric approach to NOTES. Transvaginal procedures are long established for accessing the peritoneal cavity following disinfection with antiseptic. Only experimental case series for transcolonic and transvesical approaches are described. *Conclusion*. Grade C recommendation requiring no preoperative preparation can be made for the transgastric approach. Antiseptic irrigation is recommended for transvaginal (grade C) NOTES access, as is current practice. Further human trials need to be conducted to corroborate the current evidence base for transgastric closure. It is important that future trials are conducted in a methodologically robust fashion, with emphasis on clinical outcomes and standardisation of enterotomy closure and postoperative therapy.

## 1. Introduction

Natural orifice translumenal endoscopic surgery (NOTES) represents a relatively recent development in the continuing evolution of minimal access surgery and minimally invasive surgery. Since its first published description in 2004 by Kalloo [1], NOTES has amassed a growing international body of published data dedicated to improving techniques and assessing its long term viability. Surgical access via a natural orifice rather than through a skin incision has the potential to offer great benefit over open or laparoscopic surgery, as it theoretically involves less surgical trauma and improved cosmetic result through absence of external scars, also eliminating the risk of incisional herniae. In turn, this should result in less pain, earlier return to work, and greater patient satisfaction. 

NOTES access to the peritoneal cavity has been variously described via transgastric, transcolonic, transvaginal, and transvesical approaches. There is a lack of consensus regarding the most appropriate approach; the transvaginal and transgastric are the approaches currently most commonly reported in existing literature. It is worth noting that the concept of colpotomy and transvaginal approach to intraperitoneal organs is an old one, first described by Langenbeck in 1813 for transvaginal hysterectomy [2]. One potential disadvantage of NOTES is the more difficult sterilisation of the surgical field, compared to topical skin disinfection for laparoscopic or open surgery. Transgastric and transcolonic approaches in particular involve areas normally populated by a multitude of physiological flora which, upon translocation into the peritoneum via NOTES enterotomy, have the potential to cause infection, abscess formation, and systemic sepsis. Accordingly, the Natural Orifice Surgery Consortium for Assessment and Research (NOSCAR), a key driving body in the funding and directing of NOTES research, has identified the prevention of infection as one of the key issues requiring further assessment and research [3].

At present there exist a lack of consensus and a paucity of data regarding disinfection and the prevention of infection in NOTES, and most current NOTES, research is based on experimentation in the porcine model. Experience of NOTES in humans is steadily increasing, and several case series of appendectomies via transvaginal or transgastric approaches [4, 5] and particularly cholecystectomies [5, 6] via the transvaginal approach have been published, building on initial experience with hybrid transabdominal laparoscopic/NOTES procedures [7–9]. 

The aim of this paper was to evaluate the current evidence base relating to the preparation or treatment of the access route for NOTES procedures in specific relation to postoperative infectious complications. 

## 2. Methods

### 2.1. Literature Search

A review of all the published literature on NOTES procedures reporting on infectious outcomes of access was undertaken. A comprehensive search strategy was used to identify relevant evidence according to the inclusion and exclusion criteria specified below using the following search terms: natural orifice, transgastric, transvaginal, transvesical, transcolonic, disinfection, infection, and NOTES. The search was broadened using the “related articles” function. The following mesh terms were used: “endoscopy”, “gastrointestinal”, “gastroenterostomy”, “infection”, “disinfection”, “colpotomy”, “colotomy”, and “gastrotomy”. The bibliographies of all publications were manually searched for any relevant references.

 The following electronic databases were searched: PubMed, MEDLINE (Ovid); EMBASE; Google Scholar. 

### 2.2. Endpoints

The endpoints considered for evaluation of the evidence were: (1) route of access, (2) operative intervention, (3) method of disinfection, and (4) infectious outcomes of the intervention including method of assessment.

### 2.3. Inclusion and Exclusion Criteria

Inclusion criteria included all studies considering infectious outcomes following a NOTES procedure in human or animal subjects using the transvaginal, transcolonic, transvesical, and transgastric access routes. No language restrictions were considered for review. All articles which did not refer to infectious sequelae of NOTES were excluded. Only publications relating to the highest level of evidence for each access route were described in this paper. All articles were independently reviewed as to whether they fulfilled the above-mentioned criteria.

Levels of evidence were assessed according to the Oxford Centre for Evidence-Based Medicine. Levels of Evidence guidance and recommendations were based on these levels of evidence [10]. 

## 3. Results

A total of 22 publications were included in this paper. There were 5 comparative studies and 17 case series as illustrated in Tables 1 and 2. There is currently no level 1 evidence pertaining to the most appropriate level of disinfection for any NOTES access route. The most robust evidence available in the NOTES literature relates to transgastric access with several experimental trials published. 

### 3.1. Transgastric Approach

The transgastric approach to NOTES is the most commonly investigated approach and the only one to have experimental randomised controlled trials assessing methods of infection prevention. The methods and evaluation of these trials are rather heterogeneous. Ramamoorthy et al. demonstrate in the rat model that use of PPIs can be associated with a greater rate of infection [11], in agreement with endoscopic studies in humans which have demonstrated the potential for bacterial overgrowth of the stomach with the use of PPIs [12]. This contrasts with the results reported by Eickhoff et al. suggesting that PPIs, as part of a “maximal therapy” combination including systemic antibiotics, chlorhexidine, and antibiotic orogastric irrigation, can reduce the rate of infection and intraperitoneal bacterial load after NOTES [13]. Giday et al. demonstrate a remarkable reduction in infection rates through use of povidone-iodine irrigation and systemic antibiotics compared to control in a porcine model of NOTES liver and ovarian biopsies [14]. In contrast to this, McGee et al. show no significant difference between rates of infectious complications for transgastric peritoneoscopy regardless of preparation. Pigs were randomised to receive either minimal saline, high volume saline, or antibiotic irrigation, and all 3 groups had intraperitoneal placement of a sterile foreign body. On necropsy, abscesses and obvious infection of the foreign body were seen in all 3 groups [15]. All studies used as outcome measures bacterial cultures of peritoneal and/or gastric fluid aspirates and the result of necropsy following planned euthanasia of test animals on day 14, except for Giday et al. who performed necropsy on day 7. Ramamoorthy et al. and Eickhoff et al. also assessed serial serum inflammatory markers (white blood cell count and c-reactive protein), finding no significant difference between control and trial groups.

Whilst many studies use the bacterial culturing of intraperitoneal lavage fluid as the basis of determining the presence and severity of infection, it must be noted that a positive culture result did not necessarily correlate with clinical findings in many studies [17, 18]. 

Level 3 evidence is available in the assessment of infectious complications following transgastric NOTES access. The case-controlled study by Nau et al. compared 50 patients who underwent a laparoscopic Roux-en-Y gastric bypass (RYGB) with endoscopic gastrotomy, with 50 patients who underwent laparoscopic guided NOTES RYGB (*n* = 40), or NOTES staging peritoneoscopy (*n* = 10), using disinfected but not sterilised endoscopes and no stomach preparation or irrigation. They report that in their series they found evidence of gastric to peritoneum cross-contamination (based on peritoneal aspirate cultures) in 10% of laparoscopic RYGB, 21% of NOTES RYGB, and 0% of NOTES peritoneoscopy; however, this did not correlate to clinical findings and did not affect patient management [17]. These findings are corroborated in a further study by Narula et al. [18] who report a case series of 10 patients undergoing pure NOTES transgastric peritoneoscopy without prior gastric disinfection using disinfected nonsterile equipment and no subsequent cases of clinical infection. Similarly, Dallemagne et al. [19] report a case series of 11 hybrid transgastric NOTES/transabdominal laparoscopic cholecystectomies in which they confirm contamination of the peritoneum postgastrotomy through peritoneal aspirate cultures, without clinical sequelae or consequence. 

### 3.2. Transcolonic Approach

The colon as a route of access for NOTES presents with a greater and more diverse bacterial flora than the stomach, vagina, or bladder and as a result a theoretically greater risk of peritonitis following a transcolonic procedure. Several case series have demonstrated the feasibility of this approach for procedures including laparoscopic cholecystectomy [20] and peritoneoscopy [21, 22] in porcine models. Bachman et al. [21] compared colonic irrigation with povidone-iodine and antibiotic solution with irrigation using a quaternary ammonium solution and report that both solutions were effective at significantly reducing colonic flora (by 93% and 90%, resp.), with no clinical evidence of infection at necropsy in 16 swine following transcolonic NOTES peritoneoscopy and T-tag closure. Ryou et al. [22], in their evaluation of a prototype access and closure device, describe disinfection using colonic irrigation with tap water, povidone-iodine, and antibiotic solution, with no evidence of infection of adhesions at necropsy. For the first report of human NOTES transanal rectal cancer resection using transanal endoscopic microsurgery with laparoscopic assistance, the anus and rectum were irrigated with betadine solution only without any infectious complications. However, it is important to note that extra-luminal dissection was commenced below the peritoneal reflection with the proximal rectum tied off using a purse-string suture to limit contamination [23]. The potential advantage of the transrectal route is in the relative proximity at which the colotomy can be created which may allow for very limited segmentation of the bowel for disinfection [24]. The development of new devices and overtubes to facilitate sterile transcolonic access has been reported, with Wilhelm et al. reporting successful transcolonic deployment of their device, good closure, and no signs of inflammation or infection on necropsy [25]. 

### 3.3. Transvaginal Approach

Colpotomy and the transvaginal approach to the intraperitoneal viscera have been performed for almost two centuries. NOTES represents an endoscopic extension of this technique. Disinfection of the vagina through irrigation with antiseptic such as povidone-iodine is known to produce good bactericidal results with great reduction in positive vaginal bacterial cultures [26]. As a result, the transvaginal approach for NOTES procedures is the most reported route of access in current literature, with larger case series already published for procedures ranging from laparoscopic cholecystectomies [27, 28] to NOTES-assisted right hemicolectomies [29]. The German NOTES registry has reported 572 hybrid NOTES procedures carried out on human patients, primarily cholecystectomies (448 patients) and appendicectomies (42 patients) [27]. They report a 3.3% complication rate in cholecystectomies, which includes 1 wound infection and 1 pelvic abscess requiring laparoscopic drainage, and 0% complications with appendicectomies. 

### 3.4. Transvesical Approach

Initial investigations have demonstrated the feasibility of this approach in the porcine model, including an initial series of transvesical NOTES peritoneoscopy [9, 30] with no reported infectious complications. Lima et al. [30] report a series in which NOTES peritoneoscopy is performed following cystoscopic incision of the bladder with no evidence of infection on necropsy. The same group has also published further series to include transvesical transdiaphragmatic thoracoscopy as well combined transgastric-transvesical approaches to cholecystectomy [31] and nephrectomy [32]. Initial feasibility studies have also been performed in human cadaver [33] models. A case series of 60 human patients undergoing laparoscopic prostatectomy reported minimal contamination of the peritoneum following cystotomy, with positive bacterial cultures in 5 patients, however with no clinical significance and subsequent good recovery without further intervention required [34]. 

## 4. Discussion

The current experimental and human evidence base is on a whole limited and methodologically not robust enough to allow us to draw concrete and meaningful conclusions regarding most adequate preparation and treatment of the access orifice to prevent postoperative infectious complications. 

It is obvious that evaluation of postoperative infectious complications of NOTES access as currently described in the literature are significantly related to the method of closure, and therefore studies that use different closure modalities have limited comparability. There is currently no single gastrotomy closure modality supported in the literature [27], and therefore study methodology is likely to be heterogeneous; however, for transvaginal and transcolonic access the most robust closure is likely to be suturing under direct vision. There is evidence that transvesical access does not require formal closure and will heal satisfactorily following perioperative insertion of a urethral catheter [30–32]. Furthermore, there is no reliable way of detecting clinically significant infectious complications as positive peritoneal cultures are not always clinically significant. In future human trials it is likely that evaluation will be entirely clinical as there are ethical considerations in taking peritoneal sampling postoperatively. 

It is also not known which optimal postoperative treatment of these viscerotomies is to avoid infectious complications. There is no data currently available to suggest the need for PPI or specific antibiotic cover following NOTES enterotomy, and this is likely to have an effect on infectious complication rate. 

For these reasons the interpretation of these data is complex, further complicated by the lack of controlled human trials. It is clear that for gastrotomy closure, further trials are needed to assess various modalities of prophylaxis on an individual basis. Furthermore, evaluation of the choice of equipment must also be considered—is sterile equipment required or is decontamination sufficient and is the use and further development of sterile overtubes to facilitate sterile intubation of stomach or colon indicated? These results must therefore be interpreted with care. It is difficult to draw concrete conclusions, exemplified by the study by Eickhoff et al. which does not differentiate between the individual effects of the various components of the transgastric trial therapy given and the therapy package as a whole [13], or the case-controlled study by Nau et al. which uses patients with laparoscopic gastrotomy creation as control [17]. Despite the often contradictory results from animal trials, human case series may be seen as offering level 4 evidence that no disinfection or preparation is required for the transgastric approach, suggesting a grade C recommendation that no prophylaxis is required and that further research should continue to compare other interventions to this as control.

As the transcolonic route presents perhaps the greatest potential source of bacterial translocation into the peritoneum, further study along similar lines of investigation as have been pursued for the transgastric route would be desirable. As well, the shorter route of access, compared to the traversing of oropharynx and oesophagus for transgastric access, may mean that the further development of sterile overtube devices as well as instrumentation to isolate individual colonic segments for disinfection will emerge as feasible and the most efficient way forward. Current evidence is based on animal models, and no formal recommendation can be made to recommend antiseptic irrigation for transcolonic NOTES. It is logical; however, due to the nature of the cavity that some method of disinfection is going to be necessary for this approach.

Although the transvaginal approach does not have any high level evidence supporting the most appropriate method of disinfection of the vagina preoperatively, there are large case series in the gynaecological literature which indicate that postoperative complication rates of colpotomy for tubal ligation using preoperative vaginal disinfection result in a <2% risk of infection [32, 35]. Evidence suggests that there is therefore no infectious difference between colpotomy and laparoscopic approaches for tubal ligation when the vagina is disinfected using conventional topical preparations [36]. Although there are no comparisons to control, it is generally accepted that colpotomy following disinfection of the vagina using sterile instrumentation is associated with a low and acceptable risk of bacterial contamination of the peritoneal cavity. Infectious complications are of course altered depending on the operative intervention; however, it is presumed that this risk is not dependent on preparation of the vagina. The transvaginal approach to NOTES, as a laparoscopic extrapolation of existing techniques, shows good experimental evidence of its feasibility [27, 34]. We recommend antiseptic disinfection of the vagina in accordance with current practice (grade C recommendation, based on consistent case series). There is no current evidence suggesting preferential use of either povodine-iodine or chlorhexidine.

As the urinary tract is under normal circumstances sterile, theoretically no special preparation is required for the transvesical approach. The study by McGee et al. [34] suggests that the transvesical approach is a viable option for clean access to the peritoneum requiring minimal disinfection or prophylaxis. Transvesical NOTES has yet to be performed in humans, with limited animal study-based evidence. Current experimental evidence suggests that cystotomy should be possible without specific disinfection or systemic prophylaxis (grade D recommendation).

It is important that further evaluation is conducted in a methodologically appropriate fashion. This involves specific comparisons of individual treatments/preparations of the orifice, using a standardized and identical closure modality, with objective evaluation of infectious sequelae in blinded randomised controlled trials. The results of this paper suggest that there is still a lack of experimental evidence pertaining to transrectal or transvesical access to justify implementation of human trials. Further animal evaluation is necessary for the transrectal route; however, due to the sterility of the urinary tract, it seems logical that efforts need to focus on disinfecting the external urethral orifice and surrounding areas which come into contact with operative instrumentation or the use of a sterile overtube to aid deployment of instrumentation. Due to the diameter of the urethra, current human application of this natural orifice approach has been limited.

Based on the results of this paper, we suggest that it is appropriate to embark on human randomised controlled trials in the assessment of infectious complications of transgastric access. The operative intervention must be appropriate and safe, which will likely involve a hybrid approach with closure of the gastrotomy performed laparoscopically. Outcome measures have to be objective and clinically relevant, and the levels of disinfection/sterilisation of the transgastric instrumentation or overtubes have to be standardised and clearly described as well as the postoperative treatment. As with all NOTES research, it is of vital importance that trials are carried out under IRB-approved protocols and that favourable as well as unfavourable results are disseminated through expedient publication in peer-reviewed journals as well as established NOTES registries. 

## Figures and Tables

**Table 1:** Randomised controlled trials of methods of infection prevention for transgastric NOTES procedures.

Study	Subject	Intervention	Control	Closure	Outcome measures	Conclusion
Ramamoorthy et al. [11]	30 rats	PPI prior to gastrotomy	Gastrotomy	Direct sutured	Serial WBC and CRP, day 14 necropsy and culture of peritoneum	Higher rate of peritoneal contamination and abscess formation with PPI
Buck et al. [16]	37 pigs	NOTES with mesh placement following gastric irrigation with saline and antibiotics	NOTES without mesh or endoscopy with PPI followed by laparoscopic mesh (both control groups with antibiotics and saline irrigation)	Jumbo clips	Day 14 necropsy, gastric aspirate cultures	36% NOTES mesh infection, significantly higher than laparoscopic mesh infection rate. No infection in NOTES-only control.
Giday et al. [14]	16 pigs	Systemic antibiotics and povidone-iodine gastric irrigation, NOTES liver biopsy or ovarian biopsy	NOTES biopsies without antibiotics or irrigation using disinfected but nonsterile equipment	T-bar devices	Day 7 necropsy and cultures	100% gross infection rate in control group, 0% in study group
McGee et al. [15]	19 pigs	High volume (6 L) saline or antibiotic gastric irrigation followed by NOTES peritoneoscopy and sterile foreign body placement	NOTES peritoneoscopy with 50 mL saline irrigation, sterile foreign body placement	PEG	Day 14 necropsy, gastric aspirate cultures	No differences in rate of abscess, positive culture or foreign body infection rate
Eickhoff et al. [13]	16 pigs	PPI, oral and IV antibiotics, chlorhexidine and antibiotic irrigation, NOTES gallbladder exploration and tubal ligation	Saline irrigation only, NOTES as in trial group	Plicator device	Day 14 necropsy and cultures, serial WBC and CRP	1/8 study group and 6/8 control positive cultures, significantly higher CFU load in control group

WBC: White blood cell count, CRP: C-reactive protein, PPI: proton pump inhibitor, PEG: percutaneous endoscopic gastrostomy, CFU: colony forming units.

**Table 2:** Best available evidence for preparation of orifice for transvaginal, transcolonic, and transvesical access.

Study	Access	Subject	Procedure	Closure	Infection prophylaxis	Outcome
Lehmann et al. [27]	Hybrid transvaginal NOTES/transabdominal laparoscopic	572 humans	Cholecystectomy (488), Appendix (42), other (42)	Direct sutured	Not known	3.3% complication rate for cholecystectomy, 0% for appendix. Conversion rate 4.9% overall. 1 pelvic abscess. 1 wound infection
Niu et al. [28]	Hybrid transvaginal NOTES/transabdominal laparoscopic	43 humans	Cholecystectomy (488), Appendix (42), other (42)	Direct sutured	Systemic antibiotics, local disinfection not known	No complications reported
Bachman et al. [21]	Transcolonic	16 pigs	Peritoneoscopy	Tissue Approximation System (Ethicon Endo-Surgery, Inc.)	Comparison of antibiotic/antiseptic irrigation and quaternary ammonium solution	Effective disinfection, no infection on necropsy, no difference between treatment arms
Ryou et al. [22]	Transcolonic	8 pigs	Peritoneoscopy	Prototype device	Irrigation with tapwater antibiotics and antiseptic	No evidence of infection on necropsy
Lima et al. [32]	Transvesical	8 pigs	Peritoneoscopy	None	None noted	No evidence of infection on necropsy
McGee et al. [34]	Hybrid transvesical NOTES/transabdominal laparoscopic	1 human	NOTES peritoneoscopy, robotic prostatectomy	Direct sutured	None noted	No complications reported
